# Ultra-Short Circulating Tumor DNA (usctDNA) in Plasma and Saliva of Non-Small Cell Lung Cancer (NSCLC) Patients

**DOI:** 10.3390/cancers12082041

**Published:** 2020-07-24

**Authors:** Feng Li, Fang Wei, Wei-Lun Huang, Chien-Chung Lin, Liang Li, Macy M. Shen, Qingxiang Yan, Wei Liao, David Chia, Michael Tu, Jason H. Tang, Ziding Feng, Yong Kim, Wu-Chou Su, David T. W. Wong

**Affiliations:** 1 School of Dentistry, University of California Los Angeles, Los Angeles, CA 90095, USA; frada@ucla.edu (F.W.); superliliang@126.com (L.L.); macy.macy@gmail.com (M.M.S.); wliao78@gmail.com (W.L.); dchia@mednet.ucla.edu (D.C.); mtu777@gmail.com (M.T.); truehieu@gmail.com (J.H.T.); thadyk@ucla.edu (Y.K.); 2 Department of Internal Medicine, National Cheng Kung University Hospital, College of Medicine, National Cheng Kung University, Tainan 70101, Taiwan; r4215652@gmail.com (W.-L.H.); joshcclin@gmail.com (C.-C.L.); 3 Institute of Diagnostics in Chinese Medicine, Hunan University of Chinese Medicine, Changsha 410208, China; 4 Department of Biostatistics, The University of Texas MD Anderson Cancer Center, Houston, TX 77030, USA; yan.marky@gmail.com (Q.Y.); zfeng@fredhutch.org (Z.F.)

**Keywords:** EFIRM, liquid biopsy, non-small cell lung carcinoma, usctDNA, *EGFR* mutation

## Abstract

Mutations identified in the epidermal growth factor receptor (EGFR) predict sensitivity to EGFR-targeted therapy for non-small cell lung carcinoma (NSCLC). We previously reported that Electric Field-Induced Release and Measurement (EFIRM)-based liquid biopsy could detect EGFR ctDNA with >94% concordance with tissue-based genotyping. A side-by-side comparison of concordance of EFIRM and droplet digital PCR (ddPCR) for the detection of the two front-line actionable EFGR mutations was performed with paired plasma and saliva samples from 13 NSCLC patients. Deep sequencing analysis based on single-strand DNA library preparation was employed to determine the size distributions of EGFR L858R ctDNA in plasma and saliva samples. EFIRM detected both EGFR mutations with 100% sensitivity in both plasma and saliva samples, whereas ddPCR detected EGFR mutations with sensitivities of 84.6% and 15.4%, respectively. In saliva samples, the majority of EGFR L858R ctDNA fragments detected were <80 bp. Deep sequencing analysis of ctDNA enriched for the EGFR L858R mutation revealed the significant presence of EGFR L858R ctDNA as ultra-short circulating tumor DNA (usctDNA) with the size of 40–60 bp in patient plasma and saliva. Most of usctDNAs are not amplifiable with the current ddPCR assay. Further examination using cell lines and patient biofluids revealed that the majority of usctDNAs were predominately localized in the exosomal fraction. Our study revealed the abundant existence of EGFR ctDNA in the plasma and saliva of NSCLC patients is usctDNA. usctDNA is a novel type of targets for liquid biopsy that can be efficiently detected by EFIRM technology.

## 1. Introduction

Non-small cell lung cancer (NSCLC) accounts for 85–90% of all lung cancers and is the leading cause of mortality from cancer worldwide [1]. NSCLC patients harbor the two front-line sensitizing EGFR mutations (L858R in exon 21 and deletions in exon 19), rendering them susceptible to treatment by EGFR tyrosine kinase inhibitors (EGFR-TKIs) [2]. Besides EGFR mutations, somatic genomic testing for other actionable oncogenic biomarkers including BRAF V600E mutation, ERBB2 (HER2) mutations, MET amplification as well as rearrangements of ALK, ROS1, RET, and NTRK was recommended by the guidelines from numerous professional societies [3,4,5]. Consequently, it is incumbent on physicians to determine the mutation status of EGFR and other therapeutically targetable mutations of lung cancer patients to guide precision therapy and prognostic assessment of patient health.

Liquid biopsy (LB) with non-invasive profiling of tumor-derived cell-free DNA (cfDNA), or circulating tumor DNA (ctDNA), has gained significant attention in the scientific community as a method to overcome the existing limitations of tissue biopsy techniques [6,7,8]. ctDNAs possess many tumor-associated molecular characteristics, such as genetic mutations [9,10,11] and methylation changes [12,13], which can confer specificity for tumor detection. Currently, liquid biopsy of ctDNA has already entered clinical practice. It is recommended in patients with insufficient or unobtainable tumor tissue specimen. Liquid biopsy also serves as a complement to routine tissue genotyping to identify acquired resistance mechanisms (e.g., EGFR T790M mutation) [14]. Nevertheless, the very low fraction of ctDNA relative to wild-type cfDNA background in the blood of cancer patients is an inherent challenge for liquid biopsy assays [15]. Thus, liquid biopsy analytical platforms that deliver detection sensitivity closest to biopsy-based genotyping of tumor-specific ctDNA are an unmet clinical need [16,17,18].

While liquid biopsy is an emerging field demonstrating translational and clinical utilities, much of the biological mechanisms and rationale by which tumor DNA molecules enter the bloodstream remain unclear. Recent studies using high-resolution size analysis for ctDNA and using next generation sequencing (NGS) demonstrated compelling evidence of ctDNA being shorter than the background somatic cfDNA [15,19]. In addition, the selection of short DNA fragments led to improved mutant ctDNA detection [15,20,21]. The overall size profile of plasma DNA suggests the majority of ctDNA may be derived from apoptotic/necrotic tumor cells. Exosomes found in plasma may also contain ctDNA molecules that harbor cancer somatic mutations [22,23,24], despite the source of these ctDNA not yet being resolved [25].

We have previously reported that Electric Field-Induced Release and Measurement (EFIRM) can directly detect mutant EGFR ctDNAs in plasma and saliva of NSCLC patients [26,27], including early stage disease [28]. This technology uses electric fields to enhance molecular hybridization reactions with immobilized DNA probes, followed with enzymatic signal amplification to detect ctDNA [29]. In two prospective double-blinded clinical studies, EFIRM identified the two front-line actionable EGFR mutations (L858R and exon 19del) with 84–100% sensitivity, 95–100% specificity, and 94–100% concordance with biopsy-based genotyping [26,27]. In contrast, studies to detect EGFR mutations based on PCR and NGS-based liquid biopsy technologies showed 60–80% sensitivity [30,31,32,33,34,35]. From a clinical translation perspective, EFIRM outperformed ddPCR and NGS for EGFR ctDNA detection [36]. In addition, EFIRM detects ctDNA directly, without DNA extraction nor processing. However, the mechanistic rationale of direct detection by EFIRM with a small sample volume is unclear.

To address the mechanisms underpinning the translational and clinical performance of EFIRM, we directly compared EFIRM with ddPCR on a cohort of NSCLC patients for the two front-line sensitizing EGFR mutations in biofluids (plasma and saliva). We further address the mechanistic rationale for the differential ability of ddPCR and EFIRM to detect ctDNA in saliva of NSCLC patients.

## 2. Results

### 2.1. Comparisons of EFIRM and ddPCR Detection of EGFR ctDNA in Plasma and Saliva of NSCLC Patients

A head-to-head comparison of EFIRM and ddPCR detection for EGFR L858R and exon 19 deletion mutations was performed on paired plasma and saliva samples procured from 13 NSCLC patients. The ddPCR plasma liquid biopsy assay protocol has been validated in previous reports [32,33]. All samples were blinded and randomized. Detailed information of clinical samples, genotyping results, ddPCR, and EFIRM are provided in the Supplementary Table S2.

**Figure 1:**
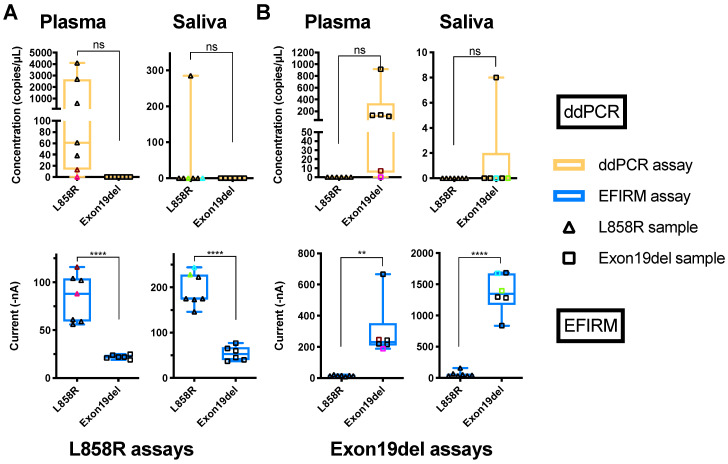
Detection of *EGFR* mutations in plasma and saliva with ddPCR and EFIRM assays. (**A**) Cell-free DNA was extracted from plasma and saliva samples from NSCLC patients. The ddPCR assay for L858R (upper row, orange) was performed with extracted cfDNA from plasma (left column) and saliva (right column). The EFIRM L858R assay was (lower row, blue) tested with different biofluid samples directly. (**B**) Results of exon 19-del detection with extracted cfDNA from different biofluids using ddPCR and EFIRM are shown in the upper row and lower row, respectively. Samples with exon 19-del mutation and L858R in *EGFR* gene identified by tissue genotyping are labeled as empty square and empty triangles, respectively. The samples with the lowest and the second-lowest copy numbers in ddPCR measurement are labeled with colors in each biofluid. The unpaired t-tests of different groups in each test were performed. ns means not significant, ** means *p* < 0.01, **** means *p* < 0.0001.

For all 13 plasma samples (tissue genotyped: six of exon 19-del and seven of L858R), ddPCR showed 11 (6/7 L858R and 5/6 of exon 19-del) out of 13 could be identified (Figure 1A,B, plasma ddPCR tests). The cut-offs of ddPCR assays for exon 19-del and L858R were five copies and one copy, respectively, based on the published literature [32,33]. Using EFIRM for direct detection of the same EGFR mutations showed 100% sensitivity and concordance (Figure 1A,B, plasma EFIRM panels). It should be noted that for plasma samples, the data from EFIRM showed less inter-patient signal variations than ddPCR. Plasmas with low copy number detection by ddPCR showed high detection signals by EFIRM (Figure 1 colored samples, comparing ddPCR with EFIRM of the same biofluid, Supplementary Table S2. This indicated that EFIRM may detect additional mutated ctDNA targets than ddPCR.

Surprisingly, for detection of EGFR ctDNA in saliva of the 13 NSCLC patients, ddPCR could only detect one of the seven L858R (14%) and one of the six exon 19-del (17%) mutations (Figure 1A,B, saliva ddPCR panels). EFIRM on the other hand, correctly identified all (13/13) seven L858R mutations and six exon 19 deletions (100% sensitivity and concordance) (Figure 1A,B, saliva EFIRM panels). These data suggest EFIRM can detect EGFR ctDNA targets that are not detectable with ddPCR in saliva.

Data also revealed strong and clustered EFIRM signals for L8585R ctDNA (200 nA) and exon 19-del ctDNA (1500 nA) in all saliva samples, a finding very different from the ddPCR detection profile in saliva (Figure 1).

One possible reason that EFIRM showed less signal variations and performed better in saliva could be that EFIRM has a higher analytical sensitivity than ddPCR. We determined the relative sensitivity/limit of detection (LOD) of ddPCR and EFIRM using mimic ctDNA samples prepared from sheared gDNA (140–200 bp) of human lung cancer cell lines HCC827 and H1975 harboring exon 19-del and L858R mutations, respectively [15]. Serially diluted, sheared gDNA samples (140–200 bp) were used for linearity and limit of detection (LOD) determination by ddPCR and EFIRM. Both ddPCR and EFIRM showed a high degree of linearity (Supplementary Figure S1). In evaluating the LOD based on the standard deviation of the response and the slope in the linear region, the LODs of EFIRM detection for exon 19-del and L858R were determined to be 7.3 and 8.9 genome equivalents (Supplementary Figure S1A,B), respectively, while ddPCR’s LODs were 8.2 genome equivalents for exon 19-del detection and 4.9 genome equivalents for L858R detection (Supplementary Figure S1C,D). Overall, the sensitivities/LOD of EFIRM and ddPCR assays were comparable for exon 19-del and L858R ctDNA detection.

### 2.2. Size of ctDNA Fragments Impact EFIRM and ddPCR Detection

Another possible factor for EFIRM’s enhanced performance in clinical samples could be the fragmentation of ctDNA. Theoretically, ddPCR and other PCR-based methods can only amplify the ctDNA fragments longer than the amplicon length (usually longer than 70 bp) and containing both primer binding sites. The fragmentation of ctDNA targets will affect the sensitivity of ddPCR. However, EFIRM is an electric field-enhanced molecular hybridization assay with a capture probe as short as 9 nt followed by a detector probe (~20 nt). The theoretical minimal physical requirements for EFIRM detection of ctDNA are 30 bp. [26,27] The differential physical requirements of target ctDNA for EFIRM and ddPCR led us to hypothesize that there are PCR-undetectable ctDNA targets in bodily fluids of NSCLC patients that can be detected by EFIRM.

**Figure 2:**
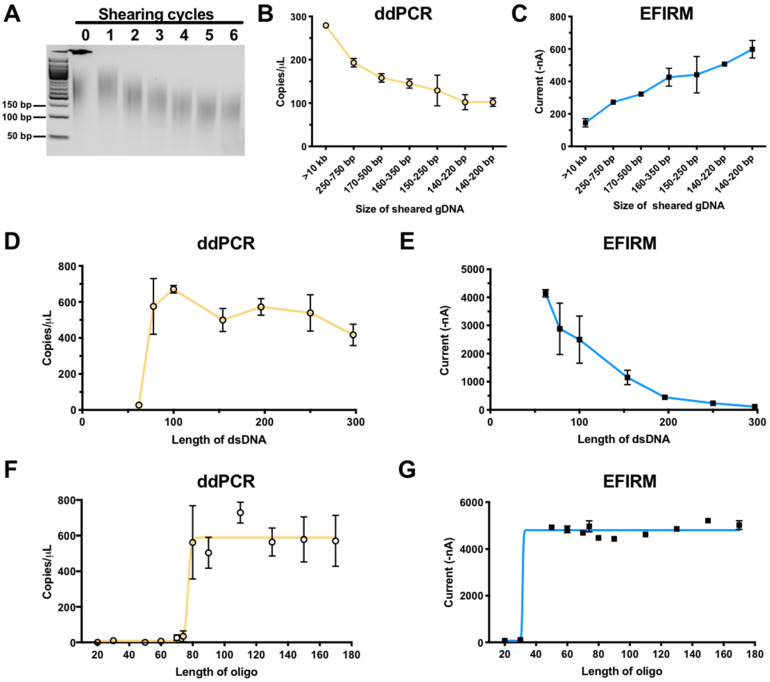
Comparison of EFIRM and ddPCR performance using sheared gDNA, dsDNA, and oligomer harboring L858R mutation. (**A**) Genomic DNA extracted from H1975 cell line was subjected to 0–6 shearing cycles with a Covaris focused ultrasonicator and was analyzed with agarose gel. Subsequently, L858R signal was detected in these samples using ddPCR (orange) (**B**) and EFIRM (blue) (**C**). A serial of PCR products of the size of 62, 78, 100, 154, 196, 250, and 297 bp with L858R mutation in similar concentrations were tested with ddPCR (**D**) and EFIRM L858R assay (**E**). Oligonucleotides (20 to 170-mer), with the L858R EGFR mutation in the center, were designed and synthesized. The ability of ddPCR (orange, **F**) and EFIRM (blue, **G**) to detect signals for those oligonucleotides was assessed. Ten pmol of each target was used for the EFIRM assay.

To test this hypothesis, we compared the performance of EFIRM and ddPCR in detecting the EGFR L858R mutation in vitro using various lengths of gDNA fragments (140 to 750 bp) from the human H1975 lung cancer cell line harboring the L858R mutation (Figure 2A). Results of ddPCR showed that the number of measurable DNA copies of L858R ctDNA decreased (from 279 to 102 copies) as gDNA was fragmented into increasingly smaller sizes (Figure 2B). This indicates that the detection of L858R by ddPCR is dependent on the fragment size of the ctDNA.

Surprisingly, EFIRM assay exhibited an opposite trend in detection sensitivity of increasingly smaller fragment sizes of ctDNA. Figure 2C showed EFIRM detection of L858R targets increased in signal intensity more than three-fold (from 145 to 599 nA) with successive shearing of the gDNA, up to six cycles to ~140–200 bp. The more fragmented the gDNA, the more efficient/sensitive the L858R fragments were detected by EFIRM, opposite to that of ddPCR.

To further confirm the impact of fragment size of the ctDNA targets, seven EGFR PCR products (sizes of 62, 78, 100, 154, 196, 250, and 297 bp) harboring the L858R mutation were tested with ddPCR (Figure 2D) and EFIRM (Figure 2E). With the same copy number of input DNA, EFIRM detecting a DNA fragment size of 62 bp showed the highest signal, and the signal decreased as the size of DNA fragment increased (Figure 2E). ddPCR, by contrast, could not detect EGFR fragments shorter than the amplicon size of 78 bp (Figure 2D).

### 2.3. Characteristics of EGFR ctDNA with L858R Mutation in Plasma and Saliva of NSCLC Patients

**Figure 3:**
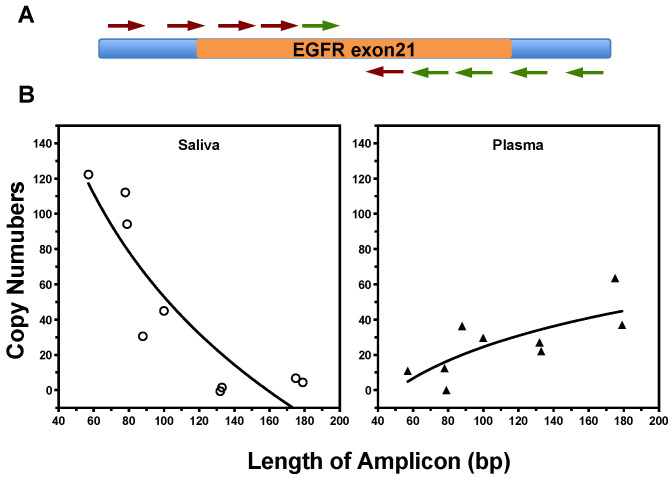
The size distribution of EGFR fragments in saliva and plasma samples from LSCLC patients (two of each biofluid). (**A**) A quantitative real-time PCR method was designed with different amplicons ranging from 55 to 180 bp, covering both sides of exon 21 of EGFR gene. (**B**) Size distribution of EGFR fragments in cfDNA samples from saliva (left) and plasma (right) was measured with qPCR test.

We next investigated the extent to which the in vitro results can be translated to the differential detection of ctDNA fragments in biofluids (saliva and plasma) from NSCLC patients. The size of cell-free EGFR DNA in patient samples was profiled by qPCR analysis with nine amplicons ranging from 55 to 184 bp, covering both sides of L858R site in exon 21 of EGFR gene (Figure 3A). The sequences of all primers pairs and the size of different amplicons are listed in the Supplementary Table S1. There is variation in the size distributions of EGFR ctDNA fragments in different biofluids (Figure 3B). The proportion of ctDNA fragments longer than 100 bp were 62.5% in plasma samples, while 96.9% of the EGFR sequences in saliva were smaller than 100 bp and 78.8% were shorter than 80 bp. The ctDNA molecules are more fragmented in saliva than plasma.

The size distribution of EGFR L858R ctDNA was further determined at single-base resolution by NGS analysis using paired plasma and saliva samples from NSCLC patients. The single-stranded DNA library generation method was used for paired-end deep sequencing, which was reported to have more effective recovery of short DNA fragments from plasma samples [37,38]. We used an enrichment procedure that enabled focus on ctDNA with the EGFR L858R mutation only.

**Figure 4:**
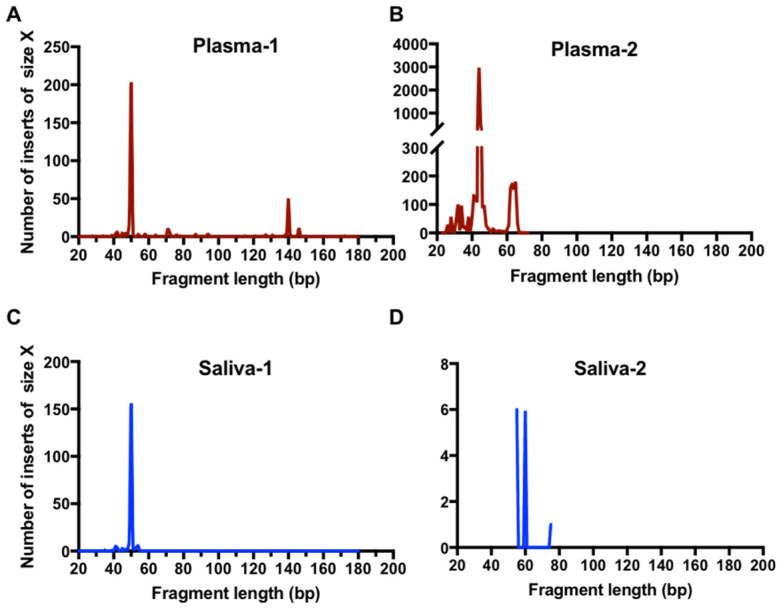
High-resolution size analysis for ctDNA harboring L858R in paired clinical samples using massively parallel sequencing. Cell-free DNA was extracted from paired plasma and saliva samples from patients. Libraries were generated with a procedure of single-stranded DNA library preparation with an L858R ctDNA enrichment step. The paired-end reads were aligned to a reference EGFR gene. The high-resolution size distribution of reads with L858R mutation from plasma samples (**A**,**B**) and saliva samples (**C**,**D**) is shown.

The results of paired-end sequencing analysis showed a large representation of extremely short DNA fragments harboring L858R mutation in all plasma and saliva tested. The size distribution of these fragments in the two paired plasma and saliva samples examined was found to be predominately between 40 and 60 bp (Figure 4). The same results were observed with the analysis of an additional two plasma samples (Supplementary Figure S2). These small ctDNA fragments were named ultra-short circulating tumor DNA (usctDNA). ctDNA harboring the L858R mutation with a size of 140–170 bp was only found in two of the four plasma samples and the amounts were very small (<5%). None was found in all saliva samples tested. The majority of the usctDNAs are not PCR amplifiable as they did not have the binding sites for both primers (Supplementary Figures S3 and S4).

**Figure 5:**
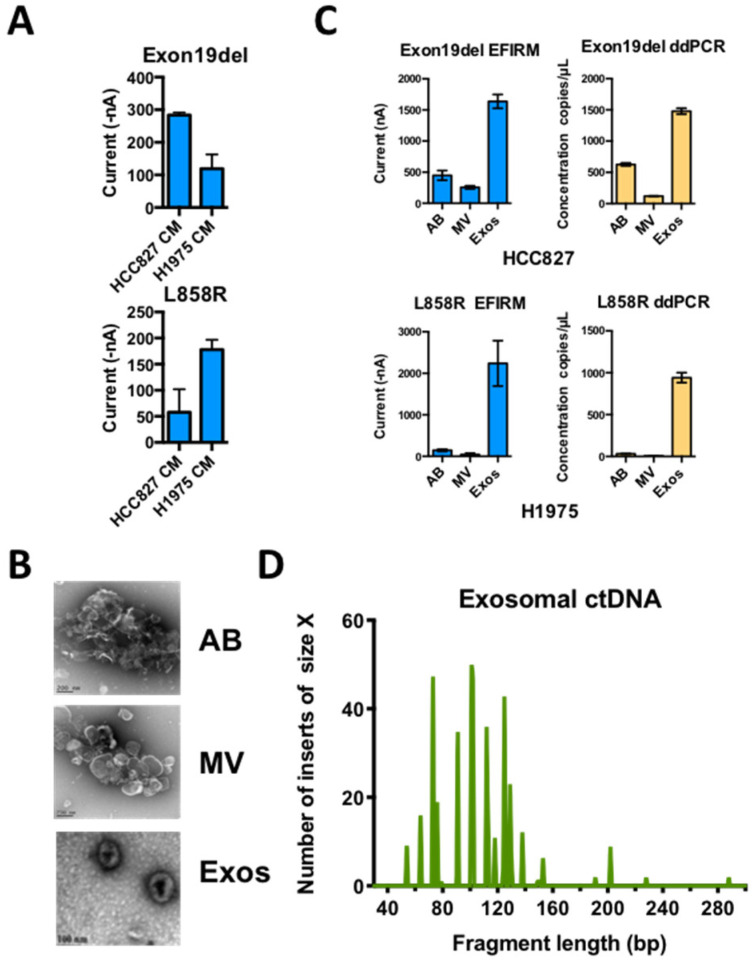
Distribution of usctDNA with mutated EGFR DNA in different extracellular vesicles from conditioned cell culture media. (**A**) Direct detection of EGFR mutations in conditioned media from H1975 and HCC827 cell culture with EFIRM assays. Apoptotic bodies (AB), microvesicles (MV), and exosomes (Exos) were isolated from conditioned media (CM) with differential ultracentrifugation method. Characterization of the vesicles by TEM (**B**). The distribution of EGFR L858R and exon 19-del ctDNA in different fractions of EVs from H1975 (**C**, lower panel) and HCC827 (**C**, upper panel) cell culture media was tested with EFIRM and ddPCR assays. The ctDNA extracted from exosome fraction was further used to generate a single-stranded DNA library. High-resolution size analysis for ctDNA harboring L858R in exosomal ctDNA was shown in (**D**).

While the proportion of longer ctDNA fragments might be influenced by the enrichment procedure with a small oligo, together with the distortion of sequential PCR amplification in library preparation, we demonstrated that the enrichment procedure was able to capture longer ctDNA fragments (Figure 5D). Thus, the usctDNA reads in sequencing data should be reflective of the in vivo stoichiometric proportions. 

### 2.4. EGFR usctDNA are Predominately Located in Exosomes

The deep sequencing data on paired plasma and saliva from the same NSCLC patient showed that the sequences of usctDNA in saliva are very similar to plasma usctDNA sequences (Figure 4, plasma-1 and saliva-1, Supplementary Figures S3 and S4). These observations led us to hypothesize that the transport of the usctDNAs is mediated by extracellular vesicles (EV). We first examined the human lung cancer cell lines (HCC827 and H1975) to determine the distribution of mutated EGFR sequences in EVs (Figure 5A). Using conditioned media from these cell lines, three extracellular vesicle fractions (apoptotic bodies, microvesicles, and exosomes) were obtained using differential ultracentrifugation and examined with transmission emission microscopy (Figure 5B). The exosome fraction from HCC827 cells was also quantified by Nanosight (Supplementary Figure S5A) and characterized by identifying the EV surface markers (CD9 and CD63) as well as by internal markers (ENO-1) using western blot analysis where the endoplasmic reticulum marker GRp78 served as a negative control (Supplementary Figures S5B and S6). EGFR L858R ctDNA was isolated from the respective EV fractions, and mutant EGFR sequences were examined with EFIRM and ddPCR assays. We found the majority of the mutant sequences, as measured by EFIRM assay, were encapsulated within exosomes (Exon 19-del: 85%; L858R: 97%) (Figure 5C). For ddPCR on the same isolated fractions, EGFR mutant sequences were also encapsulated within exosomes.

**Figure 6:**
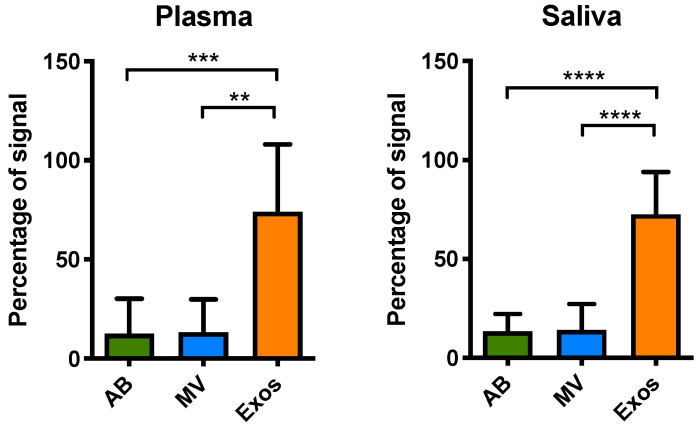
Distribution of EGFR mutations (both exon 19-del and L858R) in isolated EVs from different biofluids. Different EV fractions including apoptotic bodies (AB), microvesicles (MV), and exosomes (Exos) were isolated from the individual or pooled biofluid samples. The samples that had been previously identified as mutated and had a volume of more than 0.5 mL were chosen for isolation of extracellular vesicles (EVs). Samples with less volume were pooled for EVs isolation. We fractionated EVs from plasma (nine patients and one pooled set) and saliva (four patients and four pooled sets) with detectable EGFR L858R or exon 19-del mutations. The exon 19-del and L858R mutation detection assays were performed with EFIRM. Plasma (*n* = 10), saliva (*n* = 8). ** means *p* < 0.01, *** means *p* < 0.001, **** means *p* < 0.0001.

We proceeded to explore if EGFR mutated sequences were presented in EVs from plasma and saliva from NSCLC patients. Since usctDNA molecules were not amplifiable by ddPCR, only the EFIRM assays were used to detect the mutated EGFR sequences in EVs from all these fractions. The results showed that the highest concentration of ctDNA fragments harboring EGFR exon 19-del and L858R mutation was in exosomes (Figure 6), consistent with the findings from the cell line model.

## 3. Discussion

The success of a liquid biopsy platform inevitably depends on its sensitivity for specific targets detection. This certainly is an obstacle in the reliance on PCR or NGS-based liquid biopsy platforms. The important consideration is not only on how we detect, but more importantly, on what we are detecting. PCR-based methods have inherent disadvantages for the detection of fragmented ctDNA. We found that the physical footprint of ctDNA has profound effects on ddPCR detection (Figure 2A). With the same amount of input-sheared gDNA, the measurable copy number by ddPCR was 2.5-fold less after six shearing cycles. Our data also showed the majority of salivary ctDNA from clinical sources was shorter than 80 bp (Figure 4B saliva panel). It is currently challenging to amplify such short targets by PCR-based methods. ddPCR could only detect EGFR ctDNA in 2 out of 13 clinical saliva samples (15%) (Figure 1, saliva panels). Furthermore, most, if not all, of usctDNA detected in plasma and saliva samples by deep sequencing were not detected with ddPCR assay in this study (Supplementary Figures S3 and S4). The inability to measure usctDNA will limit the utility of PCR-based methods for liquid biopsy applications.

A significant variation in mutated ctDNA copies among NSCLC patients was observed in previous reports [39,40] and this study. It should be noted that current commercially available ctDNA extraction kits are not effective for usctDNA isolation. Together with the inability of PCR-based methods to amplify small fragmented DNAs, the actual presence of ctDNA in biofluids can be grossly underestimated. As our data demonstrated, EFIRM detected short DNA fragments more efficiently than ddPCR. In saliva samples of NSCLC patients, EFIRM assays detected the EGFR mutations in all 13 samples (100%), whereas ddPCR assays could only detect 2 of the 13 samples (15%). Our data support that the majority of ctDNA in saliva were usctDNA (<80 bp). Together with the fact that EFIRM is a direct detection of ctDNA (no extraction nor processing necessary) and detection sensitivity is inversely proportional to ctDNA fragment size, these are the triad of scientific rationale that support EFIRM’s superior detection sensitivity for ctDNA for liquid biopsy.

Currently, the turnaround times for PCR-based methods and NGS technology are approximately 2 to 3 days and 2 weeks, respectively [14]. Comparing with these established liquid biopsy methods, EFIRM assay can be performed easily in 3 h with very little hands-on time which allows a significantly faster turnaround time than NGS-based methods [41].

In this study, we identified a novel type of ctDNA, named ultra-short circulating tumor DNA (usctDNA), with sizes ~40–60 bp in both plasma and saliva from NSCLC patients. These usctDNA molecules are efficiently detected with the EFIRM technique. The mechanistic rationale that EFIRM can detect usctDNA is due to its unique detection ability based on molecular hybridization by using the capture probe of nine nucleotides that can detect fragmented DNA targets as short as 30 nucleotides. Our data showed this technology could detect small targets (<150 bp) very effectively (Figure 2E). This surface steric hindrance in EFIRM was observed as a sigmoidal curve with different lengths of target DNA fragments (Figure 2C,E). These data also indicated the limitation of the current protocol of EFIRM technology for long DNA target detection. In this study, we also demonstrated that the majority of EGFR oncogenic mutations were encapsulated within the exosomes in cell culture media of tumor cell lines and in biofluids from NSCLC patients. These data suggested that the usctDNA are packaged in the exosomes of tumor cells. These exosomes play a role in the transportation of EGFR oncogenic mutations derived from lung tumor cells to saliva through the vasculature. In our EFIRM test with clinical samples, the concentration of ctDNA with mutations in saliva is higher than in plasma (Figure 1, EFIRM panels of plasma and saliva). This indicated another route of transportation, where exosomes containing oncogenic mutations secreted from lung tumor cells can be transported via the respiratory tract into the oral cavity. A few recent studies have shown that exosomal nucleic acid can be used as a liquid biopsy source for EGFR mutations and ALK rearrangement detection in NSCLC patients [42,43]. Our results in this study also support that exosomal usctDNA can be useful for the diagnosis and prognosis of NSCLC.

Liquid biopsy is currently under active evaluation in NSCLC. Several recent investigations reported comprehensive cfDNA analysis, with plasma NGS platform showing non-inferiority compared with the standard of care tissue testing in advanced NSCLC patients [44,45,46,47]. These studies suggest that comprehensive ctDNA testing can be a primary option at the time of progression on targeted therapies. Besides in advanced cancer patients, our recent study reported that EFIRM is capable of detecting ctDNA in patients with early stage NSCLC [28]. Some other reports showed ctDNA is present in early stage lung cancer and has great potential for early lung cancer detection and screening, although the levels of ctDNA are very low [48,49]. Detecting usctDNA, which can be a novel type of target for liquid biopsy, could be very useful both in the “blood-first” scenario and in early cancer detection and screening with different sample types.

## 4. Materials and Methods

### 4.1. Patient Samples and Tissue Genotyping

Study protocols were approved by the Ethics Committee of UCLA and National Cheng Kung University Hospital (NCKUH). The IRB approval number from NCKUH is A-BR-102-098 (2014/02/18). Informed consent was obtained from all the participants included in this study according to the committee regulations. All clinical samples (plasma and saliva) were collected, and tissue genotyping was conducted with the FDA-approved EGFR RGQ PCR Kit at NCKUH. Patients with lung nodules or pleural effusion were enrolled for the study. Plasma and saliva were collected before biopsy following our standard operating procedure [26]. In total, 42 patients were enrolled, and 26 clinical samples (paired plasma and saliva samples) from 13 EGFR mutation-positive patients in their tissue genotyping were assayed for EGFR L858R and exon 19-del mutations by ddPCR and EFIRM.

### 4.2. Cell-free DNA Extraction and Quality Control

The cell-free DNA from 0.5 mL of patients’ plasma and saliva were extracted using the QIAamp circulating nucleic acid kit (Qiagen, Hilden, Germany), using the procedure as described in the manual, which had been demonstrated to provide greater extraction efficiency and higher coverage of small DNA fragments [50,51]. Total DNA from each patient sample was quantified using the fluorescence absorbance PicoGreen assay (Thermo Fisher Scientific Inc., Waltham, MA, USA).

### 4.3. Detection of Mutated Sequences Using EFIRM Assays

EFIRM is an electrochemical platform with initial mobilizing of a single allele-specific capture probe into a conducting polymer matrix on the surface of a gold electrode. The electric fields were subsequently used to facilitate allele-specific hybridization between the biological targets and immobilized DNA probes. A biotinylated detector probe with a complementary sequence adjacent to the sequence of the capture probe is further hybridized to the targets, followed by enzymatic signal amplification. Ultimately, the current of the final reaction is measured [29]. EGFR mutations in the biofluids of NSCLC patients, sheared genomic DNA (gDNA) samples, and isolated different extracellular vesicular fractions were determined by EFIRM assays, as described [26].

### 4.4. Detection of Mutated Sequences Using ddPCR

Analysis of the ddPCR data was performed using the QX100 analysis software (Bio-Rad, Hercules, CA, USA). Each sample was analyzed with 5 μL cfDNA on a QX100 digital droplet reader. Results were reported as copies of mutant allele per mL of plasma.

EGFR L858R. L858R-specific TaqMan probes/primers (Thermo Fisher Scientific Inc., Waltham, MA, USA) were used in the PCR reaction with the following cycling conditions: 95 °C × 10 min (1 cycle), 40 cycles of 94 °C × 30 s, and 58 °C × 1 min, and 10 °C hold.

EGFR exon 19 deletion. Exon 19-del-specific TaqMan probes/primers (Life Technologies, Carlsbad, CA, USA) were used in the PCR reaction with the following cycling conditions: 95 °C × 10 min (1 cycle), 40 cycles of 94 °C × 30 s and 55 °C × 1 min, followed by 10 °C hold.

Droplet digital PCR was performed as previously described on a QX100 droplet digital PCR system (Bio-Rad, Hercules, CA, USA) [32]. Results were reported as copies of mutant allele per mL of plasma. Droplet digital PCR reagents were ordered from Bio-Rad. Primer/probe mix for EGFR L858R and EGFR ex19 del was custom-ordered from Life Technologies. The primers for L858R assays with different size amplicons were listed in Supplementary Table S1.

### 4.5. Single-Stranded DNA Library Preparation and Deep Sequencing Analysis

Single-stranded sequencing libraries were prepared with an adapted protocol [52]. Libraries were prepared with 0.5–30.0 ng of DNA input and individually barcoded. The library amplification was monitored by real-time PCR and was typically terminated after 5 cycles more than Ct value. Then, hybridization capture was performed with an oligo of sequence 5′-bio-AGCAGTTTGGCCCGCCCAAAA, which was complementary to the fragments with L858R mutation. The enriched single-stranded DNA libraries were amplified in 10–12 cycles. The paired-end next generation sequencing analysis was performed with a Nextseq 550 sequencer (Illumina, San Diego, CA, USA).

### 4.6. Cell Culture and Extracellular Vesicle Isolation

The human non-small cell lung cancer cell lines HCC827 cells harboring exon 19 deletion and H1975 cells harboring L858R mutation were obtained from ATCC. HCC827 and H1975 cells were grown in RPMI-1640 medium supplemented with 10% (*v*/*v*) FBS and Pen/Strep, in an atmosphere of 95% air and 5% CO2 at 37 °C. Cells were cultured in a T75-cm2 flask until they reached 60–70% confluency. Cells were subsequently cultured in medium with exosome-free FBS for 24 h. Extracellular vesicles (EVs) released in cell culture media and three different kinds of biofluids: MPE, plasma, and saliva were isolated using a differential centrifugation-based protocol as described previously [53]. Cell-free conditioned media were centrifuged at 300× *g* for 10 min at 4 °C to remove cell debris, followed by 2000× *g* for 20 min at 4 °C to collect the apoptotic bodies. Microvesicles were collected at 16,500× *g* for 20 min at 4 °C, and exosomes were isolated at 120,000× *g* for 2 h at 4 °C. The EV pellets were washed and resuspended in ice-cold PBS. The NanoSight LM200 was used to characterize the EVs and to measure the total amount of EVs according to the manufacturer’s protocol (NanoSight, Salisbury, UK). For TEM, the EVs were mixed with 4% paraformaldehyde/1% glutaraldehyde (Merck & Co., Kenilworth, NJ, USA). The samples were loaded onto carbon-coated formvar grids (Ted Pella Inc., Redding, CA, USA), washed by MQ water, and negatively stained with 0.2% uranyl acetate (Merck & Co., Inc.) for 3 min. The samples were then air-dried and examined with a JEM-1400 Transmission Electron Microscope or a JEM-2100F CS STEM Electron Microscope (JEOL, Peabody, MA, USA). For the patients’ biofluids, exosomes were collected by filtering the supernatant through 0.2 µm pores and concentrating to a final volume of 100 μL using a Vivaspin Turbo 15 concentrator.

## 5. Conclusions

In this study, we demonstrated the presence of usctDNA fragments containing EGFR mutations in plasma and saliva of NSCLC patients. In saliva, they are principally of the ultra-short configurations. The majority of usctDNA molecules are localized in the exosome fraction of biofluids and cannot be detected by PCR-based analysis. Missing these important biological indicators in assays will have an impact on sensitivity and result in the underestimation of the copy number of ctDNA in clinical samples. The EFIRM platform has the advantage by nature of its capability of detecting usctDNA, which EFIRM’s requirement of small volume of clinical samples may be attributed to. Perfecting the detection sensitivity of liquid biopsy to non-invasively detect tumor-specific ctDNA is of paramount clinical importance as it impacts on early detection, risk assessment, screening, diagnosis, and personalized/precision medicine.

## Supplementary Materials

The following are available online at https://www.mdpi.com/2072-6694/12/8/2041/s1, Table S1. The sequences of primers used for ddPCR assays in Figure 3D and qPCR assay (Figure 4B); Table S2. The tissue genotyping information, ddPCR results and EFIRM test results of paired plasma and saliva samples from NSCLC patients; Figure S1. Comparison of the limit of detection (LOD) of EFIRM and ddPCR for detection of EGFR exon19del and L858R mutations; Figure S2. High-resolution size analysis for ctDNA harboring L858R in plasma samples using massively parallel sequencing; Figure S3. Mapping results of represented ctDNA with L858R mutation from plasma samples; Figure S4. Mapping results of represented ctDNA with L858R mutation from saliva samples; Figure S5. Quantification of exosomes isolated from the cell culture medium; Figure S6. The original Western Blots images for Figure S5B.
