# Supplementary material for: Ultra-Short Circulating Tumor DNA (usctDNA) in Plasma and Saliva of Non-Small Cell Lung Cancer (NSCLC) Patients
Source: Cancers (Basel). 2020 Jul 24;12(8):2041. doi: 10.3390/cancers12082041 (PMC7464208; doi:10.3390/cancers12082041)
Supplement: Supplementary file 1 [file cancers-12-02041-s001.pdf]

Supplementary Materials

Ultra-Short Circulating Tumor DNA (usctDNA) in Plasma and Saliva of Non-Small Cell Lung Cancer (NSCLC) Patients

Feng Li [1,†,*], Fang Wei [1,†], Wei-Lun Huang [2], Chien-Chung Lin [2], Liang Li [1,3], Macy M. Shen [1], Qingxiang Yan [4], Wei Liao [1], David Chia [1], Michael Tu [1], Jason H. Tang [1], Ziding Feng [4], Yong Kim [1], Wu-Chou Su [2,*] and David T.W. Wong [1,*]

Table S1. The sequences of primers used for ddPCR assays in figure 3D and qPCR assay (figure 4B).

Primer ID	Sequence	Primer Pair	Amplicon Size (bp)
L858 F1	ACACCGCAGCATGTCAA	F1+R1	55
L858 F2	GGAACGTACTGGTGAAAACAC	F1+R2	83
L858 F3	TGGCAGCCAGGAACGTA	F1+R3	105
L858 F4	AGGGCATGAACTACTTGGAG	F1+R4	137
L858 F5	CCCATGATGATCTGTCCCTC	F1+R5	184
L858 R1	CCGCACCCAGCAGTTTG	F2+R1	72
L858 R2	CCTCCTTCTGCATGGTATTCT	F3+R1	81
L858 R3	CTAAAGCCACCTCCTTACTTTG	F4+R1	126
L858 R4	TGGTCCCTGGTGTCAGGA	F5+R1	168
L858 R5	ATCCTCCCCTGCATGTGTTA	CCRF+R6	62
L858 R6	ATTCTTTCTCTTCCGCACCC	CCRF+CCRR	78
L858 CCR F*	GCAGCATGTCAAGATCACAGATT	CCRF+R3	100
L858 CCR R*	CCTCCTTCTGCATGGTATTCTTTCT	F4+R2	154
		F5+R2	196
		F5+R4	250
		F5+R5	297

* Primers from previous paper [1]

Table S2. The tissue genotyping information, ddPCR results and EFIRM test results of paired plasma and saliva samples from NSCLC patients.

			Plasma Tests				Saliva Tests			
**Sample ID**	**tissue genotyping**	**TNM**	**ddPCR Exon 19-del assay**	**Exon 19-del EFIRM**	**ddPCR L858R assay**	**L858R EFIRM**	**ddPCR Exon 19-del assay**	**Exon19del EFIRM**	**ddPCR L858 assay**	**L858R EFIRM**
UCLA-088	EX19Del	T4N3M1b	2	246.8	0	23.7	0	1398.0	0	40.4
UCLA-097	EX19Del	T3N3M1b	12	215.3	0	21.3	0	837.4	0	65.1
UCLA-099	EX19Del	T1bN3M1b	2	665.6	0	24.9	2	1684.4	0	45.5
UCLA-100	EX19Del	T4N3M1b	4	219.3	0	21.9	0	1675.0	0	36.8
UCLA-103	EX19Del	T3N2M1b	30	243.0	0	18.6	0	1282.7	0	59.8
UCLA-091	EX19Del	T4N3M1b	0	188.1	0	22.3	0	1296.0	0	77.1
UCLA-082	L858R	T3N3M1b	0	15.5	591	55.8	0	158.2	76	223.1
UCLA-101	L858R	T3N3M1b	0	13.8	10	61.2	0	32.1	0	145.7
UCLA-111	L858R	T4N3M1b	0	14.3	419	59.1	0	34.9	0	174.9
UCLA-137	L858R	TxNxM1a	0	21.5	2	116.0	0	66.7	0	228.0
UCLA-138	L858R	T3N3M1b	0	15.7	6	103.9	0	50.9	0	244.3
UCLA-143	L858R	T4N3M1b	0	17.0	88	102.2	0	44.4	0	175.3
UCLA-152	L858R	T4N2M1b	0	8.4	0	88.1	0	31.9	0	173.3

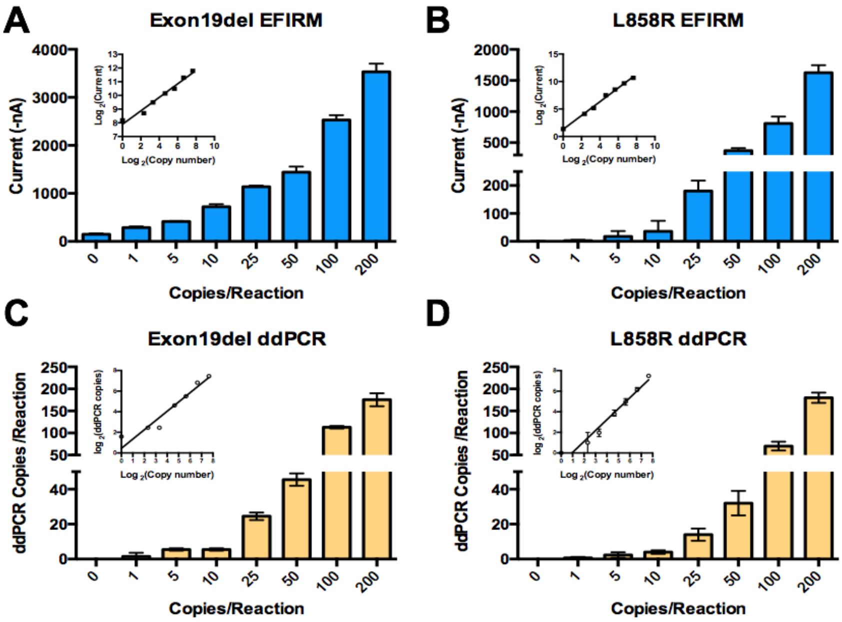

Figure S1. Comparison of the limit of detection (LOD) of EFIRM and ddPCR for detection of EGFR exon19del and L858R mutations. Mimic ctDNA samples were generated by shearing the gDNA from HCC827 cell (harboring ex19del) and H1975 cell (harboring L858R) to the final size of 140–200 bp. The total copy number of mutated DNA targets were determined using ddPCR assays. The serially diluted samples were used for relative LOD determination by EFIRM (**A** and **B**) and ddPCR (**C** and **D**). Both platforms showed a high degree of linearity (small panels). The LOD was determined based on the standard deviation of the response and the slope in the linear region.

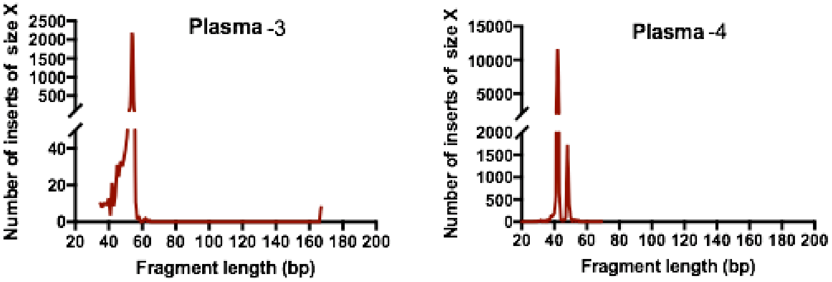

Figure S2. High-resolution size analysis for ctDNA harboring L858R in plasma samples using massively parallel sequencing.

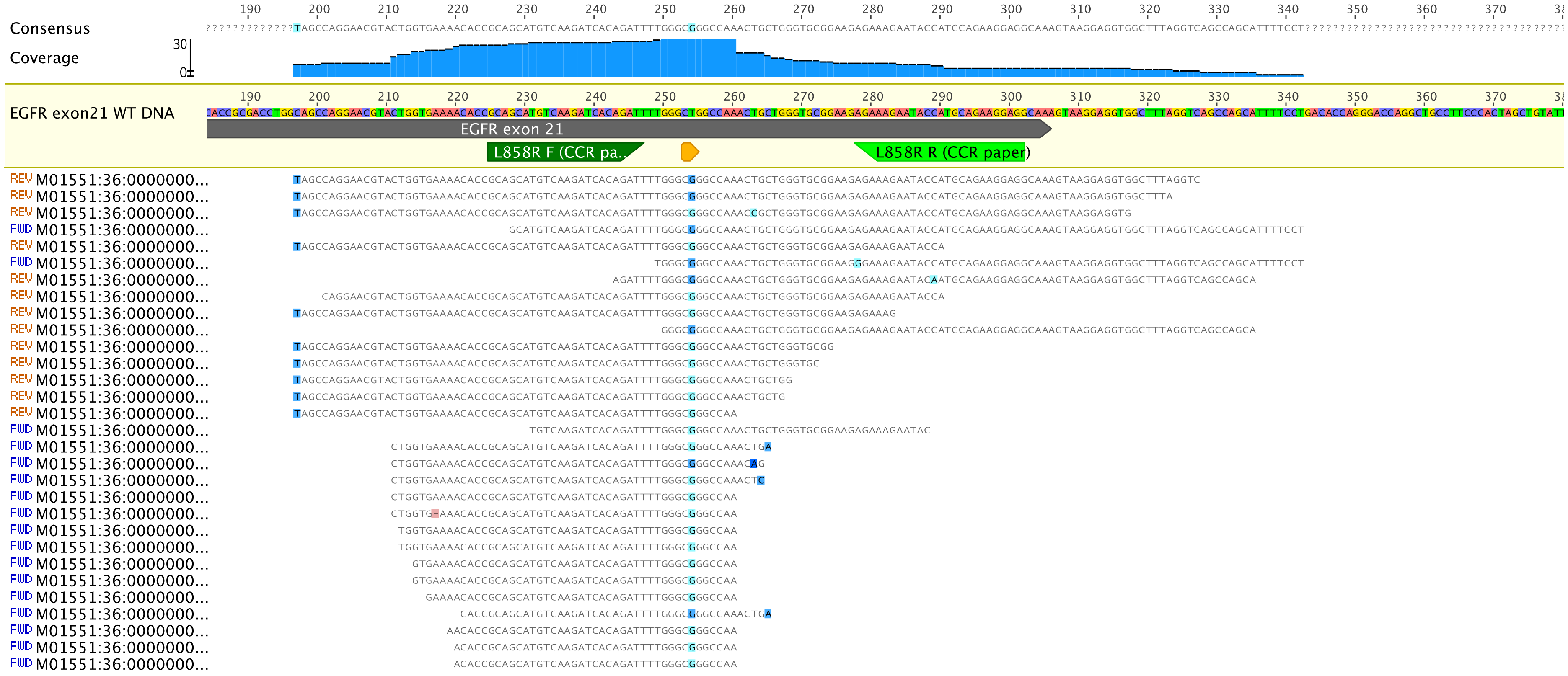

Figure S3. Mapping results of represented ctDNA with L858R mutation from plasma samples.

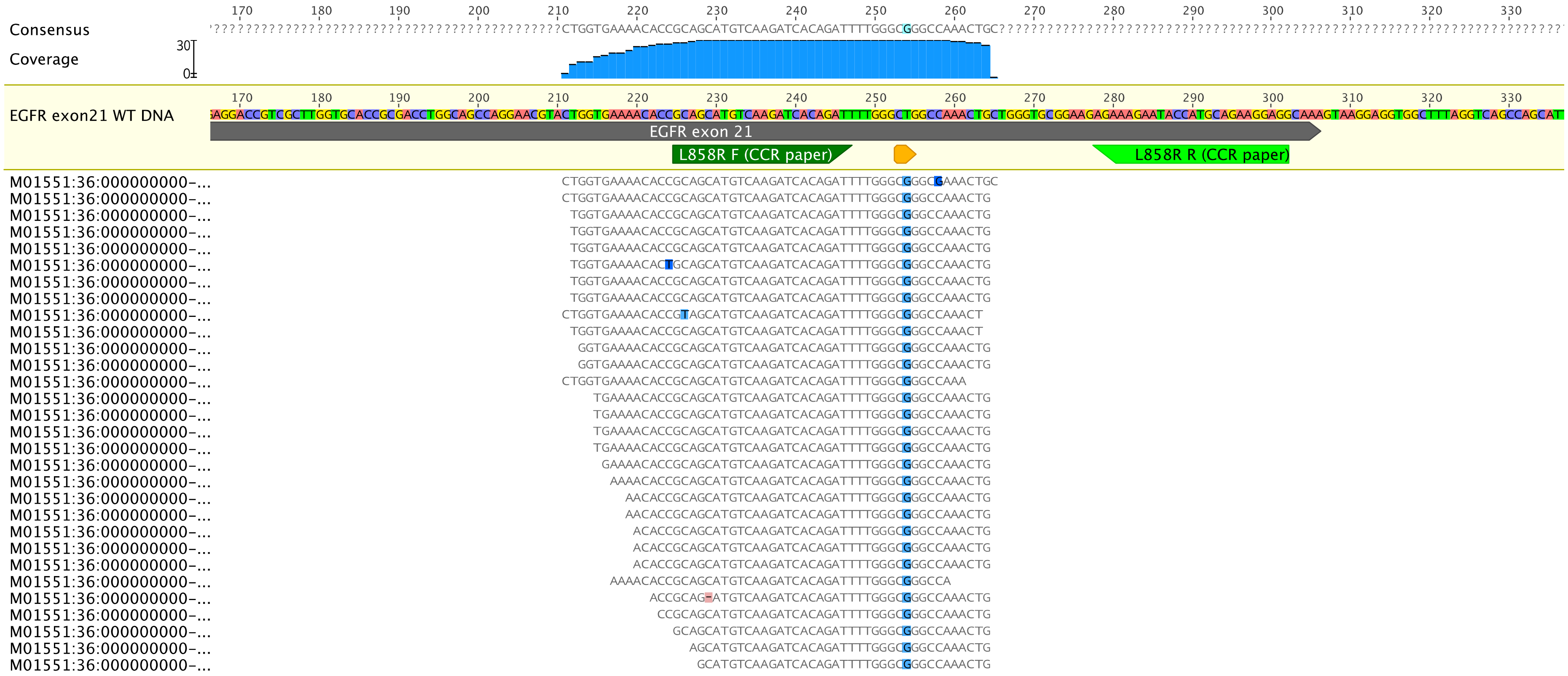

Figure S4. Mapping results of represented ctDNA with L858R mutation from saliva samples.

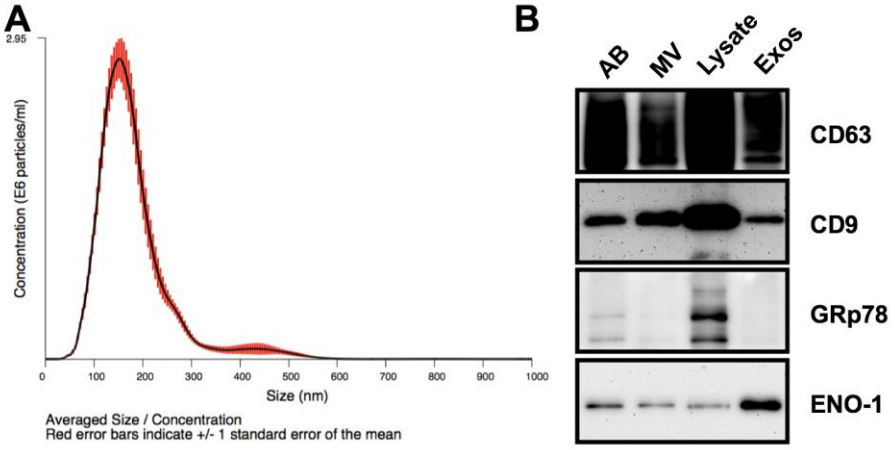

Figure S5. Quantification of exosomes isolated from the cell culture medium. (**A**) Verification of exosomes using Nanosight. (**B**) Characterization of HCC827 derived EVs by western blot analysis with the EV external surface markers (CD9 and CD63) as well as by internal markers (ENO-1) where the endoplasmic reticulum marker GRp78 served as a negative control. All original Western Blots figures can be seen in Figure S6

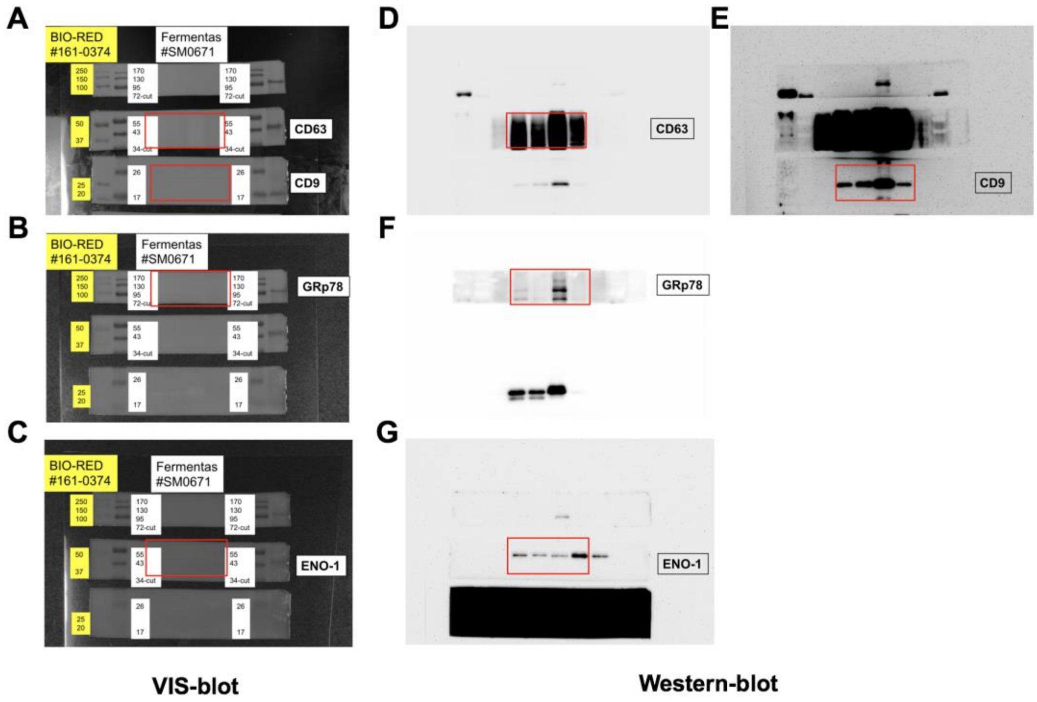

Figure S6. The original Western Blots images for figure S5B. The whole blot membrane was sliced for different antibody detection. The same membranes were used and re-probed with different antibodies after stripping to use the EV samples effectively. The whole blot images under visible light were listed on the left panel (**A**, **B** and **C**). Two different molecular weight markers were labeled on the blots. The right panel showed corresponding images after probing with different antibodies (**D**, **E**, **F** and **G**). The cropped regions for Figure S5B were identified with red frames.

References

1. Pu, D.; Liang, H.; Wei, F.; Akin, D.; Feng, Z.; Yan, Q.; Li, Y.; Zhen, Y.; Xu, L.; Dong, G.; et al. Evaluation of a novel saliva-based epidermal growth factor receptor mutation detection for lung cancer: A pilot study. *Thorac. Cancer* **2016**, *7*, 428–436, doi:10.1111/1759-7714.12350.

© 2020 by the authors. Licensee MDPI, Basel, Switzerland. This article is an open access article distributed under the terms and conditions of the Creative Commons Attribution (CC BY) license (http://creativecommons.org/licenses/by/4.0/).
